# *Chlamydia trachomatis *infection and sexual behaviour among female students attending higher education in the Republic of Ireland

**DOI:** 10.1186/1471-2458-9-397

**Published:** 2009-10-29

**Authors:** Emer O'Connell, Wendy Brennan, Martin Cormican, Marita Glacken, Diarmuid O'Donovan, Akke Vellinga, Niall Cahill, Fionnguala Lysaght, Joan O'Donnell

**Affiliations:** 1 Department of Public Health, Health Service Executive, Western Area, Galway, Ireland; 2 Department of Microbiology, University College Hospital Galway (UCHG), Galway, Ireland; 3 Department of Bacteriology, National University of Ireland, Galway, Ireland; 4 Department of General Practice, National University of Ireland, Galway, Ireland; 5 Student Health Unit, University of Limerick, Limerick City, Ireland; 6 Student Health Unit, National University of Ireland, Galway, Ireland; 7 Faculty of Public Health Medicine, Royal College of Physicians in Ireland, Dublin, Ireland

## Abstract

**Background:**

There are no prevalence data on *Chlamydia trachomatis *relating to female students attending higher education available for the Republic of Ireland. This information is required to guide on the necessity for Chlamydia screening programmes in higher education settings. This research aimed to determine the prevalence of and predictive risk factors for *Chlamydia trachomatis *genital infection among female higher education students in Ireland.

**Methods:**

All females presenting during one-day periods at Student Health Units in three higher education institutions in two cities in the Republic of Ireland were invited to participate. Participants completed a questionnaire on lifestyle and socio-demographic factors and provided a urine sample. Samples were tested for *C. trachomatis *DNA by a PCR based technique (Cobas Amplicor, Roche). To examine possible associations between a positive test and demographic and lifestyle risk factors, a univariate analysis was performed. All associations with a p value < 0.05 were included in a multivariate logistic regression analysis.

**Results:**

Of the 460 sexually active participants 22 tested positive (prevalence 4.8%; 95% CI 3.0 to 7.1%). Variables associated with significantly increased risk were current suggestive symptoms, two or more one-night stands and three or more lifetime sexual partners. The students displayed high-risk sexual behaviour.

**Conclusion:**

The prevalence of *C. trachomatis *infection and the lack of awareness of the significance of suggestive symptoms among sexually experienced female students demonstrate the need for a programme to test asymptomatic or non-presenting higher education students. The risk factors identified by multivariate analysis may be useful in identifying those who are most likely to benefit from screening. Alcohol abuse, condom use, sexual behaviour (at home and abroad) and, knowledge of sexually transmitted infections (STIs) (including asymptomatic nature or relevant symptoms) were identified as target areas for health promotion strategies. These strategies are needed in view of the high-risk sexual activity identified.

## Background

*Chlamydia trachomatis *is an obligate intracellular bacteria associated with sexually transmitted infection (STI). Symptoms may include discharge and dysuria although a high percentage (70%) of infections are asymptomatic particularly in women [1]. Untreated *C. trachomatis *infection may progress to pelvic inflammatory disease in approximately 40% and is a leading cause of female infertility [1,2]. Unlike England's National Chlamydia Screening Programme (NCSP), Ireland has no national programme for detection of *C. trachomatis *infection in those who do not present seeking care [3].

*C. trachomatis *is a notifiable disease in Ireland. The number of notifications increased from 245 in 1995 to 1,278 in 2003. After 2004 when legislation requiring laboratory notification came into effect, the number further increased to 3,144 in 2006 [4]. *C. trachomatis *is the second most commonly notified STI in Ireland (32% of all notifications), after ano-genital warts [4].

The majority of notified cases (70%) of infection are among 20-29 year olds. People aged 0-19 years account for 14% of notifications and 30-39 year olds account for 13% [4]. European studies of students attending higher education (males and females) have found prevalences of *C. trachomatis *infection ranging from 0 to 12% [5-11]. England's NCSP had a positivity rate of 5.5% for female students attending a variety of educational levels [3]. Studies from North America in the 1990s and the 2000s found prevalence rates in female students attending higher education ranging from 2.3 to 10% [12-17]. Studies on this female population from other parts of the world show prevalence rates between 1.1 to 10.6% [18-22]. No prevalence data relating to female students attending higher education are available in Ireland. This research aimed to measure this to inform planned Chlamydia screening programmes.

## Methods

### Ethics

The study protocol received ethical approval from the Clinical Research Ethical Committee of University College Hospital Galway and Merlin Park Hospital, Galway and from the University of Limerick Research Ethics Committee.

### Screening sites

Female students attending three Student Health Units (SHU) in three higher education institutions in two cities were invited to participate in the study. These institutions included two universities and one institute of education, all in the Republic of Ireland. (Higher education is the educational level following the completion of a school providing a secondary education, such as a high school or a secondary school. It includes universities, colleges and institutes of technology).

All female students attending a screening site on designated screening days between October 2004 and March 2005 were approached. This amounted to 71 screening days in total i.e. an average of 27 per screening site.

### Recruitment and screening process

A different method of recruitment was used in each setting. One involved the healthcare provider (nurse/doctor) advising the female attendees of the screening programme after their consultation had concluded. A second option (due to the frequently lengthy waiting periods in one setting), involved advising students of the study while waiting for their appointment. This was done mainly by the receptionist. If students were willing to consider participating in the study, they were asked to read the study information leaflet attached to the study pack. This described the study and suggested that, if interested in participating, one could complete a self-administered questionnaire while waiting for an appointment with the healthcare professional. The third option involved the nursing staff referring students to the principal investigator (medical doctor) either after the student's main consultation had taken place or while the student awaited an appointment. In all recruitment scenarios, if the student then indicated an interest in participating, the significance of a positive chlamydia test and the subsequent management of same were explained to them by a healthcare professional.

### Data and sample collection

Written consent, a self-administered questionnaire and a urine sample were gathered from each participating student. Demographic details and use of the oral contraceptive pill were gathered on non-participants (Table 1). These demographic details included age, faculty and year in higher education.

**Table 1:** Comparison of non-participants to participants

**Variables**	**Categories**	**Non-participants**	**Participants**	**Significance of difference**
N		73	450	
Mean age in years (std)		21.1 (3.6)	20.6 (2.4)	ns
Years in higher education (std)		2.3 (1.3)	3.0 (1.5)	0.001
		N (%)	N (%)	
Higher Education Institution	A	15 (20.5)	114 (25.3)	ns
	B	29 (39.7)	180 (40.0)	ns
	C	29 (39.7)	156 (34.7)	ns
Ireland is not usual country of residence		4 (5.5)	28 (6.2)	ns
Current user of oral contraceptive pill		39 (55.6)	269 (59.8)	ns

ns = non-significant, std = standard deviation

The questionnaire consisted mainly of the dichotomous yes/no option and the forced choice question. Open-ended questions were also utilised. The questionnaire included demographic questions such as medical card status (A medical card provides access for those on low incomes to all public health services) (Table 2). Also included were questions about sexual health and sexual lifestyle (casual Sex was described as when a person has sex with another person more than once but not within a relationship).

**Table 2:** Description of participants

**Variables**	**Details**	**Participants**
Number		450
Age (years)	Mean	20.6
	Median	20.0
	St. D	2.4
	Range	17-34
		N (%)
Usual country of residence	Ireland	422 (93.8)
	All other countries	28 (6.2)
Home setting	Urban	192 (42.7)
	Rural	257 (57.1)
	Missing	1 (0.2)
"Marital" Status	Single	358 (79.6)
	Other	85 (18.9)
	Missing	7 (1.6)
Graduate Status	Undergraduate	383 (85.1)
	Postgraduate	56 (12.4)
	Missing	11 (2.4)
Year in higher education	1st Year	93 (20.7)
	2^nd ^Year	97 (21.6)
	3^rd ^Year	88 (19.6)
	4^th ^Year	90 (20.0)
	5^th^/> year	81 (18.0)
	Missing	1 (0.2)
Faculty	Humanities	69 (15.3)
	Medicine & Health	24 (5.3)
	Science	84 (18.7)
	Commerce/Business	123 (27.3)
	Engineering/Computing	42 (9.3)
	Other	83 (18.4)
	Missing	25 (5.6)
Education	Part-time	12 (2.7)
	Full-time	438 (97.3)
Job	No	176 (39.1)
	Full-time	12 (2.7)
	Part-time	262 (58.2)
Medical card	Yes	93 (20.7)
	No	357 (79.3)

### Exclusion criteria

Students presenting with symptoms suggestive of a STI, requesting assessment for STIs, or who had received antibiotic treatment in the previous two weeks were excluded. Regarding the urine sample the student must not have urinated in the previous two hours and a first void urine sample was necessary.

### Specimen and case management

Specimens were tested by Roche Amplicor PCR according to the manufacturer's instructions (Cobas Amplicor-operational manual 5/2003 Revision 3.0. Roche Molecular Systems, Inc., Branchberg, NJ 08876 USA) with initial positive samples confirmed by retesting. Participants who tested positive were contacted by telephone and referred to a doctor in the Student Health Unit for further care and contact tracing.

### Statistical analysis

Data were analysed using SPSS for Windows (Chicago Ltd. Version 13). A variable, "number of sexually active years" was created from current age and age of first episode of sexual intercourse to reflect the duration of sexual activity. The variable "suggestive symptoms" consisted of one or more of the following: abnormal vaginal discharge, bleeding post-coital/intra-menses, non-menses lower abdominal pain, dysuria/frequency of micturition.

The three higher educational institutions were compared for sociodemographic factors. To examine possible associations between a positive test and demographic and lifestyle risk factors, a univariate analysis was performed. All associations with a p value < 0.05 were included in a multivariate logistic regression analysis. To minimise the impact of highly correlated lifestyle factors, a correlation analysis was performed and this guided multicollinearity in the model.

## Results

Of the 690 female students attending the Student Health Units on the study days 617 (89.4%) agreed to participate. The 73 students who declined to participate did not differ from participants by age, higher education institute attended, country most lived in or use of oral contraceptive pill (OCP) (Table 1). However participants were generally attending higher education longer then non-participants (3.0 years vs. 2.3 years, p = 0.001).

A urine sample was provided by 496 (71.8% of students) students, however 36 of these students indicated that they were not sexually active and 10 diagnostic test results were invalid. Of the 450 sexually active students for whom a valid test result was returned, 22 were positive for a prevalence rate 4.8% (95% CI 3.0 to 7.1). Assuming all non-participants and invalid samples were not infected the minimum estimate of the prevalence of *C. trachomatis *infection can be calculated to be 3.2%.

Significant differences between the three institutions existed for marital status, study subject, rural background and medical card ownership. These variables were controlled for in the multivariate analysis.

The median age of the participants was 20, with a range of 17 to 34 years (Table 2). Ninety-four percent of the study population lived in Ireland for the majority of their lives. Eighty percent were single or not living with a partner. Eighty-five percent were undergraduates with an even spread in each year in higher education education.

### Sexual Health

Two hundred and fifteen (48%) participants reported having sexual intercourse (SI) at least once per week. The mean age for first sexual intercourse was 17.6 years (median 17). The mean number of lifetime sexual partners was 4.7 (range 1-40, median 3). One or more "one-night stands" was reported by 203 (45%) participants and 157 (35%) experienced one or more "casual sexual relationships".

Two hundred and twenty two (49%) of the participants had one or more new male sexual partners in the previous 12 months with a range from 1 to 11 (mean 1.1, median 1). Unprotected intercourse with a new male partner was reported by 180 (40%). Consumption of alcohol by self (19%) or by the male partner (12%) was the most frequently given reason for not using a condom. Two hundred and ten participants (47%) had never carried a condom when having sex with a new male partner- the main reason given was that these episodes of sexual intercourse were not planned. Sex with a new partner while travelling outside Ireland in the previous year was reported by 51 (16%) students.

Condom use was the current method of contraception for 222 (49.6%). The "morning after pill" had been used by 184 (41%) participants and 104 (23%) had used withdrawal as a method of contraception. A previous STI was reported by 42 (9.3%) of whom 8 (1.8%) reported previous *C. trachomatis *infection. Of these students with an STI, 32% (15) were not advised about partner notification.

Univariate analysis showed infection with *C. trachomatis *was significantly associated with (1) number of lifetime partners, (2) number of "one-night stands", (3) suggestive symptoms, and (4) sexual intercourse with a new partner and no condom used.

Due to high correlations between several variables, different models of multivariate analysis were constructed. Two final models were retained; model 1 allows for international comparison with similar use of variables (Table 3). Model 2 is presented for practical and screening purposes (Table 3). The fit of the models was assessed with the Hosmer and Lemeshow Goodness of Fit. A residual analysis was performed and did not show up major outliers influencing the results.

**Table 3:** Prevalence and multivariate associations of Chlamydia infection with demographic, behavioural, and clinical factors for female higher education students†

				**Model 1**		**Model 2**	
	**Total**	**N (%) Chlamydia positive**		**OR**	**95% CI**	**OR**	**95% CI**
Higher Education Institution							
A	114	4 (3.5)		ref		ref	
B	180	13 (7.2)		2.4	0.8-8.0	2.1	0.6-7.3
C	156	5 (3.2)		1.0	0.3-4.0	1.1	0.3-4.5
Number of lifetime sexual partners							
1	125	3 (2.4)	1-2	ref			
2	79	1 (1.3)					
3	54	5 (9.3)	≥ 3	3.6	1.4-9.5*		
≥ 4	187	13 (7.0)					
SI with a new partner and no condom used							
No	263	8 (3.0)					
Yes	180	14 (7.8)					
Number of one-night stands							
0-1	324	12 (3.7)				ref	
≥ 2	125	10 (8.0)				2.7	1.0-7.7*
Number of sexually active years							
≤ 1	35	0 (0.0)	per year	0.8 ^$^	0.7-1.1	0.9^$^	0.8-1.2
1-2	79	3 (3.8)					
2-3	91	4 (4.4)					
3-4	91	9 (9.9)					
4-5	64	4 (6.3)					
≥ 5	90	2 (2.2)					
Have suggestive symptoms							
No	341	12 (3.5)		ref		ref	
Yes	109	10 (9.2)		2.5	1.0-6.3*	2.5	1.0-6.9*

† Model 1 allows for international comparison. Model 2 assists identification of high-risk students for screening purposes.

ref: reference category

* p value significant

^$ ^for every year increase

The odds of Chlamydia infection was strongly associated with suggestive symptoms (OR 2.5, CI 1.0-6.2, p = 0.05) (Model 1, Table 3) and also with having three or more lifetime sexual partners (OR 3.6 CI 1.4-9.5, p = 0.01).

In model 2 (Table 3) the number of lifetime sexual partners is replaced by the number of one-night stands, which has significance (p = 0.006) for two or more one-night stands (OR 2.7, CI 1.0-7.7).

## Discussion

This study is the first Irish multi-site study of the prevalence of *C. trachomatis *infection in women who do not present seeking care. The prevalence of 4.8% among female students attending higher education was broadly comparable with international experience. This prevalence may support a screening strategy as models have shown screening to be cost effective at prevalences of 3.1-10.0%, and cost saving at a prevalence as low as 1.1% [23].

Risk factor findings are also comparable to international data; the odds ratio of 3.6 for being Chlamydia positive with three or more lifetime sexual partners is very similar to that recorded by Imai for Japanese female students (OR 3.4) with the same sexual history [19]. Also the odds ratio of 2.5 for being Chlamydia positive if have current symptoms corresponds closely to the odds ratio of 2.1 for American students with current symptoms [16].

In our study we decided to report on strict cut off values for risk factors to make risk assessment by a clinician practical. Risk assessment is increasingly important in this current economic recession and in the midst of the pandemic H1N1 2009, as both clinician's time and laboratory resources are increasingly limited. Two or more one-night stands and three or more lifetime sexual partners significantly increase the odds of a positive result. These sexual history questions could be asked during a consultation to assist in identifying patients at high-risk for Chlamydia infection and thus maximising the positive yield from the samples submitted.

The median age of 17 years for the first episode of sexual intercourse is the same as that found among women aged 18-24 in the 2004 "Irish Study of Sexual Health and Relationships" (ISSHR) [24].

However our students displayed generally more high-risk behaviour than was reported in the sexual health survey of Irish undergraduate students at Trinity College Dublin; they were more likely to have a one-night stand (45% vs 30%) and more likely to use "withdrawal" as a contraceptive method (8% vs. less than 1%) [25]. They also had more sexual partners (34% vs 16% of female students having 5 or more).

The risk associated with travel abroad (of sex with a new partner) was lower in our study at 16%, than the 32% of British medical students that Finney reports [26]. However as the setting of a holiday carries an increased potential for risky sexual behaviour such as unsafe sex added with exposure to different sexual networks, travel advice to students should cover safer sex and the prevalence of STIs in the area to be visited [27].

Risk behaviour was often associated with an increased use of alcohol as many students in our study blamed alcohol for not using a condom for sex with a new partner. It is likely that alcohol also enabled the lack of planning for sexual intercourse, which led to non-carrying of condoms. This negative influence of alcohol should be emphasised in future health campaigns.

The low percentage of students with a past history of Chlamydia infection (1.8%) indicates that many positive cases are not tested, possibly due to the asymptomatic nature of Chlamydia. As the majority of female Chlamydia infections are asymptomatic this implies that a high proportion of infections are not detected with the potential for adverse health outcomes. In addition 24% of the students had suggestive symptoms at the time of the fieldwork and were not presenting with these, thus indicating a low level of understanding of potential STI symptoms. It is likely that these symptoms were mild and not impacting on the students' daily activities. However this is worrying as having suggestive symptoms significantly increases the risk of a positive test. Though contradictory to the idea of screening, programmes need to target both asymptomatic and non-presenting symptomatic students. A diversified range of screening strategies as used by the NCSP (e.g. web-based requests for testing kits, "pee-in-pot" days on campus) would reach more students especially those who do not attend health care settings [3].

Also of concern 32% (15) of students with an STI report that they were not advised about partner notification; a lack of support services in the SHUs (at the time of this study) for this public health work is a likely reason. (Particular efforts were made during this study to provide robust partner notification to the identified positive cases). To minimise spread of infection, future screening programmes need to provide adequate support services such as a community based health advisor.

## Limitations

Higher education students are not representative of the general population. Students attending Student Health Units may not be representative of all students as some students may not seek any medical care and others may seek care at their family doctor or elsewhere. Voluntary recruitment may also have favoured participation by students already concerned regarding STIs. Participants were generally attending higher education longer then non-participants and thus may have either had more concerns due to a possibly longer sexual history or may have been more comfortable with screening in the Student Health Units.

## Conclusion

The prevalence of infection at 4.8% is comparable to international data and within a suggested range of cost effective prevalence levels. Risk factors identified by multivariate analysis were current suggestive symptoms, two or more one-night stands and three or more lifetime sexual partners. These observations may be useful in identifying those who are most likely to benefit from screening.

This study also showed significant levels of high-risk sexual activity amongst female students attending higher education. Alcohol misuse, condom use, sexual behaviour (at home and abroad) and knowledge of STIs (including asymptomatic nature or relevant symptoms) are health topics, which should be included in STI management/prevention campaigns at higher education institutions.

Based on this pilot study, a more comprehensive study to assess the value and feasibility of a national screening programme for *C. trachomatis *infection in Ireland is now underway.

## Competing interests

The authors declare that they have no competing interests.

## Authors' contributions

EOC designed the study, obtained ethical consent, recruited participants, collected data, analysed data and drafted the manuscript. WM carried out laboratory testing and participated in design and helped draft the manuscript. MC participated in the design, monitored and guided laboratory procedures and helped draft the manuscript. MG participated in design and coordination. DOD participated in the design and coordination and helped draft the manuscript. AV performed the advanced statistical analysis and helped draft the manuscript. NC participated in data collection and recruitment. FL participated in data collection and recruitment. JOD participated in the design and coordination and analysis. All authors read and approved the final manuscript.

## Pre-publication history

The pre-publication history for this paper can be accessed here:


